# Dynamics of Innate Immunity in SARS-CoV-2 Infections: Exploring the Impact of Natural Killer Cells, Inflammatory Responses, Viral Evasion Strategies, and Severity

**DOI:** 10.3390/cells14110763

**Published:** 2025-05-22

**Authors:** Juan C. Batista, Rodrigo DeAntonio, Sandra López-Vergès

**Affiliations:** 1 Department of Research in Virology and Biotechnology, Gorgas Memorial Institute of Health Studies, Panama City 0816-02593, Panama; juanbatista@css.gob.pa; 2 MD-PhD Program on Clinical and Biomedical Research, Faculty of Medicine, Universidad de Panamá, Panama City 0830-00929, Panama; 3 Caja de Seguro Social, Panama City 0816-06808, Panama; 4 Centro de Vacunación e Investigación CEVAXIN, Panama City 0830-00507, Panama

**Keywords:** SARS-CoV-2, COVID-19, severity, innate immune response, NK cells, cytokines, inflammation

## Abstract

The COVID-19 pandemic, caused by SARS-CoV-2, has had a profound impact on global health, with nearly 800 million cases reported in the Americas alone. The clinical presentation of the disease is highly variable, with approximately half of all patients experiencing severe symptoms. This variability confounds the complex interplay between immune responses and disease severity. Severe cases are often characterized by elevated levels of inflammatory cytokines. Over 88% of COVID-19 patients have multiple comorbidities; factors such as age and pre-existing conditions further modulate immune responses and contribute to the severity of the disease. While some studies have reported differences in cytokine profiles between severity groups, larger, well-designed cohorts are needed to clarify these relationships. Natural Killer cells, which are critical for the innate immune response against SARS-CoV-2, are often impaired and contribute to immune exhaustion. In addition, SARS-CoV-2 evades innate immune defenses through accessory proteins that inhibit interferon signaling and exacerbate cytokine storms and inflammation. This integrative review aims to synthesize findings from 2020 onward and provide insights into the innate immune responses induced by SARS-CoV-2 and their contributions to disease pathogenesis. Understanding cytokine dynamics, NK cell behaviors, and viral immune evasion strategies is critical for advancing therapeutic approaches.

## 1. Introduction

**Figure 1:**
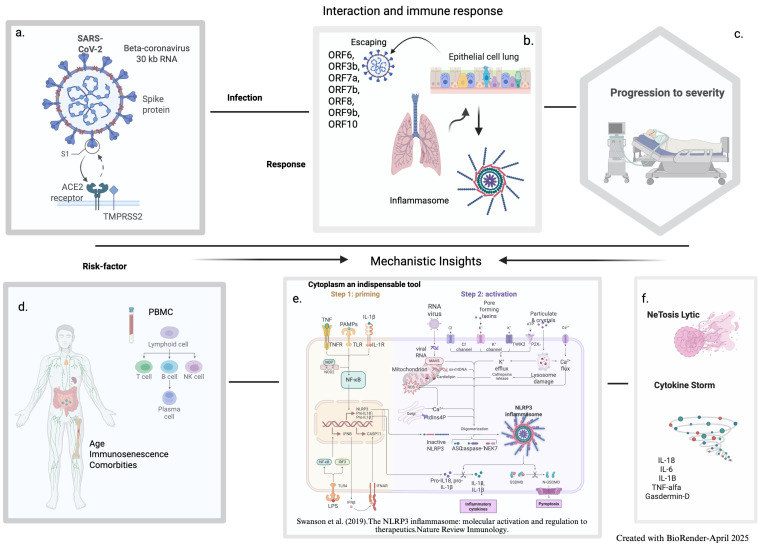
The interaction between SARS-CoV-2 and the innate immune response can define disease severity. (**a**): The structure of the SARS-CoV-2 virus particle with its 30 kb RNA viral genome and Spike proteins on its surface. The Spike protein (S1) binds to the host cell’s ACE2 receptor, facilitated by the enzyme TMPRSS2, allowing the virus to enter the target cell. (**b**): SARS-CoV-2 escaping the immune response of the infected lung epithelial cells, marked by proteins such as ORF6 and ORF3b, interacting with the inflammasome. The lungs are illustrated between the infected cells and the inflammasome, highlighting the effect of the immune response on their functions. (**c**): Hospitalized patient on a ventilator, symbolizing the progression to severe COVID-19. (**d**): Host factors (age, immunosenescence, and comorbidities) impact the human lymphatic system and immune cells (PBMC). (**e**): The NLRP3 inflammasome activation process occurs in two steps: priming triggered by TNF, IL-1*β*, and PAMPs, leading to NF-kB activation and NLRP3/pro-IL-1 β expression and activation driven by viral RNA, mitochondrial stress, K^+^ efflux, and lysosome damage. NLRP3 activates caspase-1, converting pro-IL-1 β and pro-IL-18 into active cytokines and triggering pyroptosis (inflammatory cell death) via GSDMD pores [8]. (**f**): Lytic NEtosis and cytokine storms drive severe inflammation in immune dysregulation.

The severe acute respiratory syndrome coronavirus 2 (SARS-CoV-2) virus causes widespread immune–inflammatory disorders and a dysregulated host response to infection. It is a beta coronavirus belonging to the *Coronaviridae* family with an approximately 30 kb non-segmented RNA genome [1]. The coronavirus contains four major structural proteins: the nucleocapsid (N) protein, the transmembrane (M) protein, the envelope (E) protein, and the spike (S) glycoprotein, as well as non-structural proteins (NSPs), including accessory and replicase proteins [2,3]. Transmembrane protease serine 2 (TMPRSS2) facilitates the binding of S to the ACE2 receptor on the surface of epithelial cells, which promotes viral entry [2]. The expression of the ACE2 receptor in the ciliated cells of the superficial epithelium of the nasal cavity indicates an initially localized infection. Later, as the infection progresses, SARS-CoV-2 spreads throughout the respiratory tract, often causing respiratory illness, although the virus can also infect other systems and organs, as shown in Figure 1 [4,5]. However, not all infections result in hospitalization, and patients with coronavirus disease 2019 (COVID-19) may present with a spectrum of symptoms, ranging from mild cases to severe conditions that require medical attention or can result in death. At the same time, some children may have mild symptoms such as a runny nose, cough, or fever and recover quickly at home [6]. COVID-19 cases are categorized as mild, moderate, severe, or critically ill. Those with pneumonia-related imaging findings are classified as moderately severe cases. Patients experiencing respiratory distress—defined as a respiratory rate ≥ 30/min, an oxygen saturation ≤ 93% on room air, or a PF ratio ≤ 300 mmHg—are considered severe cases [7].

Outcomes depend on associated variables that can affect the complexity of the virus and the host’s physiology and immune system [9]. On the viral side, the route of viral transmission, the initial viral load at infection, and the mutations of the SARS-CoV-2 variants influence disease severity and the immune response, including inflammation and the virus’s ability to escape [10]. On the other hand, the health status of hosts and their response to SARS-CoV-2 are highly variable and have a significant impact on the development and outcome of the infection. Various epidemiological and clinical studies, especially those characterizing COVID-19 patients with moderate to severe acute respiratory distress syndrome (ARDS), indicate that older individuals with underlying health conditions are more susceptible to severe illness [11].

The innate immune response represents the first line of host defense against viral infection, and the interaction between the immune system and the host is critical to limiting and eradicating infection [12]. Initially, the infected alveolar epithelial and endothelial cells in the respiratory system trigger the production of proinflammatory cytokines [2,13]. Although inflammation and cytokines represent different aspects of the body’s immunological response to viral infection, they are closely related in the context of COVID-19 [14,15]. Excessive inflammation leading to a cytokine storm has been suggested as a key driver of COVID-19 immunopathogenesis [5,15,16] and its progression to the more severe fatal form. Due to these immune response dysregulations, patients may experience varying degrees of clinical severity.

This review will describe the current knowledge on the roles of cytokines, inflammation, and innate immune cells, focusing on Natural Killer cells and their influence on the overall immune response, pathogenesis, disease progression, and severity of COVID-19. By examining studies on the interactions between the innate immune system and SARS-CoV-2, this review aims to provide insights into the mechanisms underlying immune dysregulation, identify knowledge gaps that further research can address, and determine potential therapeutic targets to improve outcomes for patients with severe COVID-19 and other coronavirus-related diseases.

## 2. Methodology of the Literature Review

A systematic literature search was performed using the PubMed database with a general literature search framework (Supplementary Figure S1) on the inflammation and immune response to infection and its dysregulation during COVID-19. In addition, studies focusing on how SARS-CoV-2 evades the immune response in COVID-19-affected patients were included. The bibliographic database search included studies published between 2020 and 2024, which were updated for the discussion with the main 2025 findings to ensure the most recent findings were included.

### Selection Process

Upon locating the articles identified by the search strategy, the titles and abstracts from each were screened and evaluated by two independent reviewers based on eligibility, recency, and inclusion and exclusion criteria. It is important to elaborate on the aspects used to select the analyzed articles as recommended by the PRISMA guidelines. Eligibility criteria for study inclusion and full-text analysis were as follows: (1) only original research articles; (2) articles in the English language, as it is the predominant language in the scientific literature and facilitates universal access to sources; (3) free direct access to the full text; (4) published within the last 5 years; and (5) focused on human subjects or samples (Table 1). The articles were also selected based on the availability of sufficient methodological detail for comparison, with an emphasis on articles evaluating innate immune responses to COVID-19 (natural infection), reporting immunological markers relevant to innate responses (e.g., cytokine levels), and Natural Killer (NK) cells.

Systematic reviews and meta-analyses were excluded, as this review aimed to focus on original or primary research that contributed novel findings to the understanding of inflammation, cytokines, NK cells, and innate immune responses in COVID-19. Studies that met the inclusion criteria underwent a full-text review. We intentionally opted not to include studies such as meta-analyses due to the substantial heterogeneity in study designs, populations (e.g., age ranges from neonates to the elderly), methodologies for measuring immune response, and outcome reporting. This heterogeneity would prevent meaningful data pooling without introducing bias or reducing interpretability. These criteria were designed to ensure scientific relevance and comparability within the scope of our narrative synthesis.

We divided the analyzed articles into two groups: primary and secondary (Supplementary Figure S1). For the primary articles, we used the results of our initial search. The initial search focused on innate immunity to coronavirus infection using broad terms such as “innate immunity”, “coronavirus”, “SARS-CoV-2”, “cytokine”, and “inflammation”. This approach allowed us to cover a wide range of relevant studies from the literature. Although specific terms were not included from the outset, the search found studies that provided an integrative view of the immune response to the coronavirus. This provided a comprehensive foundation for the analysis of the intricate interactions of the innate immunity in the context of COVID-19. The second group of studies focused on the importance of Natural Killer cells and their role in the early immune response to COVID-19. These studies explore how NK cells, along with other components of the innate immune system, such as cytokines, interact with the SARS-CoV-2 virus. Our second specific research did not focus on other innate immune cells.

By prioritizing original research articles, this review sought to capture specific data and findings that directly investigated the variability of inflammation and immune responses related to SARS-CoV-2 infection. We aimed to identify potential trends or fundamental patterns while also examining inconsistencies or contradictions in the research. These discrepancies, when present, were considered potential indicators of areas requiring further investigation. As part of our literature synthesis to understand the current state of knowledge and provide a comprehensive view of a particular topic by synthesizing existing research, particularly in areas with original articles with discrepancies, we used existing reviews, expert opinions, theoretical perspectives, and synthesized knowledge as complementary literature for the discussion and future research directions (Supplementary Figure S1). For this complementary literature, recent articles and systematic reviews published during the first months of 2025 were also included to provide up-to-date information. The figure summarizing the main information was created with BioRender.com accessed in 2024, last access in April 2025.

We adopted a structured narrative synthesis, grouping studies by key themes. Additionally, we added a Supplementary Materials summarizing cohort sizes, age ranges, sex distribution (when available), and main methods used to support transparency and allow readers to assess comparability and observed trends across studies.

This methodology strengthens the accuracy of the review’s objectives by combining broad terms with strict criteria to achieve a balance between comprehensiveness and relevance. The focus on NK cells constitutes a distinctive analytical subgroup. Clear visualization and complementary secondary literature are used to contextualize the findings.

## 3. Results

### 3.1. Results of the Bibliographical Search

**Figure 2:**
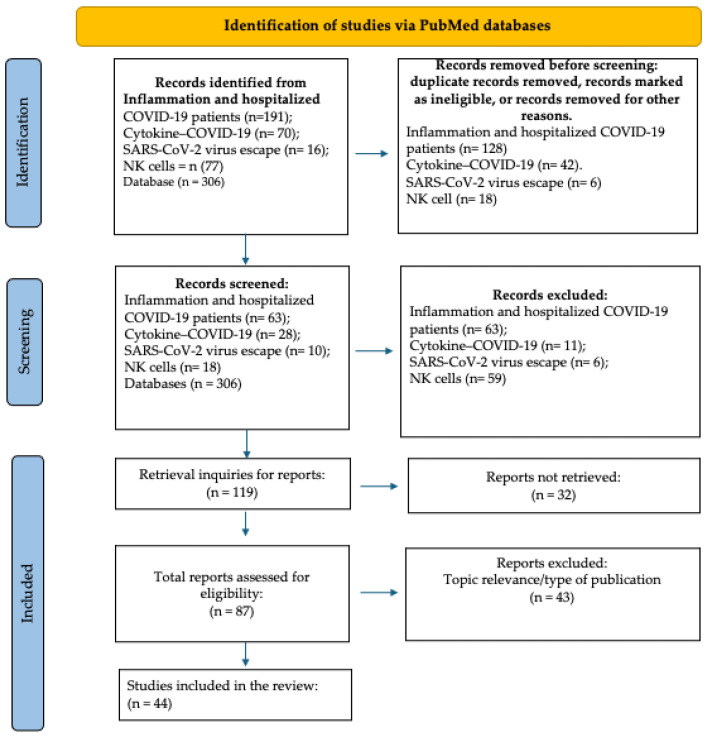
Literature review flow diagram.

During the first literature research focused on broad terms, 6585 documents were identified by the browser; however, after applying the inclusion and exclusion criteria, a total of 894 studies were identified in the first phase of our review (Supplementary Table S1). After this identification phase, documents were screened and filtered to finally include 44 articles in this review analysis (Figure 2, Supplementary Table S1). After a detailed lecture on the selected articles, the main results for each article were summarized in a table (Supplementary Table S2) for analysis, comparison, and discussion in the review.

### 3.2. Main Scientific Findings from the Analyzed Literature

#### 3.2.1. Inflammation Induced by SARS-CoV-2 Infection and COVID-19 Severity

The complex interaction between the innate and adaptive immune systems plays a key role in the immunological response to SARS-CoV-2 infection. Both systems are essential for the immune response to COVID-19 and in managing the disease. Immune response is pivotal in determining the severity of illnesses and is crucial for the growth, healing, and preservation of damaged tissues. The induction of the immune response begins as soon as the virus enters the host and interacts with the receptors on target cells. In infections, alterations in host cell physiology lead to inflammasome activation and NF-κB signaling, triggering a cytokine storm, autophagy, and cell death [17]. These alterations are linked to the modification of epigenetic patterns caused by SARS-CoV-2 and genetic variations in genes such as ACE2 and TMPRSS2, which affect cellular gene expression and immune reactions [18,19]. By using pattern recognition receptors (PRRs) such as Toll-like receptors to identify the presence of viruses, the innate immune system offers the first line of defense. This detection triggers the activation of innate immune responses through the induction of antimicrobial genes and the release of inflammatory cytokines and interferons, which prevent viral replication and attract immune cells to the infection site [9,18]. The activation of Toll-like receptors and the induction of strong type I and III interferon (IFN) responses in acute viral infections result in the transcription of hundreds of IFN-stimulated genes. Type I IFN in humans consists of IFN-α—which is important for activating NK cells, differentiating T cells, and inhibiting the proliferation of T cells—and IFN-β—which acts as an inhibitor of viral replication in infected cells and plays a defensive role in uninfected cells. These processes lead to the production of proinflammatory cytokines and chemokines (such as IL-6, TNF-α, MCP-1, and MIp-1α) and promote the robust differentiation of Th1 cells, which destroy infected target cells.

The balance between the role of type I IFN in the antiviral response and the induction of an inflammatory state is complex. Some studies have shown that IFN-α inactivation or IFB dysfunction is an important contributor to disease severity in SARS-CoV-2 infection, whereas another study showed that high levels of IFN-α were associated with a higher risk of respiratory failure [20,21]. This should be taken into account when determining which patients could benefit from IFN treatments, as it was linked to protection before the viral peak but with pathogenesis during the inflammatory response. During severe COVID-19-related ARDS (acute respiratory distress syndrome), specific proinflammatory cytokines such as IL-6, IL-8, and IL-10 appear at higher levels [5,7]. Clinical investigations have demonstrated that respiratory impairment is a significant predictor of responsiveness to cytokine blocking therapy, given the pivotal role that IL-1 and IL-6 play in triggering a hyperinflammatory response and pulmonary damage. More specifically, early intervention with IL-1 and IL-6 inhibitors has shown promise in improving outcomes for COVID-19 patients with hyperinflammatory disease and severe pneumonia [22].

The interplay between these pathways and the presence of growth factors such as PDGF-AA (Platelet-Derived Growth Factor-AA), PDGF-AB-BB (two isoforms), FGF-2 (Fibroblast Growth Factor 2), and soluble CD40L in the plasma of infected patients contributes to vessel remodeling, angiogenesis, and the pathogenesis of severe diseases, highlighting the intricate relationship between infection, immune responses, and host cell alterations [23,24].

Monocyte recruitment to the region of inflammation is facilitated by two chemokines, MCP-1 [25] and CCL2 [7]. After localized nasal airway infection, as the SARS-CoV-2 infection progresses through the respiratory system, infected alveolar epithelial and endothelial cells trigger the production of proinflammatory cytokines such as IP-10/CXCL10, TNF-alpha, and IL-6, leading to neutrophil recruitment and cell death through processes such as NETosis [2]. NETosis is a unique form of cell death in neutrophils, in which they release their DNA to form neutrophil extracellular traps (NETs) composed of DNA, histones, and various proteins, which trap and neutralize pathogens [26,27]. This process results in further complications such as platelet activation, thrombus formation, loss of junctional integrity, and edema [28,29]. A significant characteristic of a dysregulated immune response is the release of proinflammatory cytokines, leading to a phenomenon known as a cytokine storm, which can drastically alter cellular immunity. This hyperactive immune response is characterized by the overproduction of inflammatory cytokines such as IL-6, IL-8, and IL-10, which are notably elevated in severe cases of COVID-19, contributing to immune dysregulation and increased disease severity [30,31]. Cytokine storms can lead to lymphocytopenia, reduced lymphocyte counts, and an elevated neutrophil-to-lymphocyte ratio, all of which are markers of a poor prognosis in critically ill patients [32,33].

Some characteristics of inflammasomes have been identified as key signaling platforms responsible for detecting pathogenic microorganisms. These intricate mechanisms are crucial for maintaining the delicate balance between the inflammatory response and the immune system, making the cytoplasm an indispensable tool for identifying pathogenic organisms that produce and secrete proinflammatory compounds [34]. The activation of NLRP3 by SARS-CoV-2 occurs through the binding of Spike glycoprotein to the cell surface receptor ACE2, leading to viral internalization and replication. The interaction among viral proteins, viroporins, and NLRP3 facilitates the assembly of the inflammasome through oligomerization, interaction with ASC (Apoptosis-associated Speck-like protein), containing a Caspase Activation and Recruitment Domain (CARD), and cleavage of caspase-1. This process results in the maturation and release of IL-18, IL-6, IL-1B, TNF-alpha, and Gasdermin-D, leading to a pore-forming protein that incites pyroptosis (a type of inflammatory programmed cell death) [35]. Recent data suggest that Galectin-3, a β-galactoside-binding lectin, contributes to SARS-CoV-2-induced hyperinflammation by promoting NLRP3 inflammasome activation and enhancing the secretion of IL-1β and IL-18, thereby exacerbating tissue damage and disease severity [35,36]. SARS-CoV-2 infection produces an uncontrollably activated NLRP3 inflammatory protein in macrophages and epithelial cells, which not only promotes viral replication but also exacerbates the inflammatory response. This excessive stimulation is directly related to the severity of COVID-19, as shown by elevated NLRP3 levels in critically ill patients [37,38,39].

In this context, modular therapies such as NLRP3 inhibitors (e.g., MCC950) or blocking the purinergic receptor P2X7, a key regulator of inflammation, are promising strategies. Although MCC950 aims to reduce inflammatory pain, P2X7 inhibition may also reduce viral replication and tissular damage. These findings, supported by recent studies [40], highlight the urgency of investigating interventions that restore the immunological equilibrium in patients with COVID-19 [37].

The cytokines produced by the innate immune response can shape the cytokine response from adaptive immunity, which also plays a role in infection resolution and immune memory, or in exacerbating pathogenesis, leading to severity or chronicity. Proinflammatory Th1 responses are constrained by Th2 cytokines and typically result in the resolution of infection and immunity. In COVID-19, the dysregulation of Th1 and Th2 responses leads to cytokine syndrome, where high levels of diverse cytokines drive a positive feedback loop of pathogenic inflammation [41]. At least 11 cytokines (GRO-a, IL-1RA, IL-6, IL-8, IL-10, IP-10, MIG, FGF-2, IL-5, MDC, and MIP-10) have demonstrated statistically significant differences between the early and late stages of COVID-19 infection in several investigations [7,41]. Understanding the inflammatory response and the role of cytokines during infection could help identify biomarkers for prognostics. Patients who experienced severe and serious COVID-19 demonstrate an increase in IL-6 [42] at the peak of their symptoms [6,34]. Research shows that in patients with COVID-19, IL-8 concentrations reliably indicate disease severity and provide more accurate clinical predictions compared to IL-6 [43]. Examples such as urinary tract infections and measuring IL-8 in urine samples yield superior diagnostic precision over blood tests [44]. In cases of pediatric ARDS, elevated IL-8 levels serve as important indicators of poor outcomes, including an increased mortality risk and prolonged ventilator dependence [45]. In terms of severity, sensitivity, and specificity, IL-8 outperforms IP-10 (interferon-gamma-inducible protein 10) and MDC as biomarkers during early-stage SARS-CoV-2 infection. However, IP-10 remains an early-stage biomarker for predicting disease severity. Taken together, these studies highlight IL-8’s effectiveness as a diagnostic and prognostic tool across multiple infectious and inflammatory diseases, serving as a highly valuable biomarker for COVID-19.

Emerging SARS-CoV-2 variants continue to influence the host immune response and inflammatory profile. The JN.1 variant, a recent descendant of the SARS-CoV-2 BA.2.86 lineage, has attracted recent attention due to its unique mutational pattern and ability to alter the host immune response. Its potential effect on the synthesis of key chemokines, including IL-8 and IP-10, which are known markers of severity in COVID-19 cases, is especially significant. The genetic features of this variant provide insights into JN.1’s immunological role, although it remains unclear how it might encourage the synthesis of these proinflammatory chemicals [46].

#### 3.2.2. Immune Cells and COVID-19

The hematological system was significantly impacted by COVID-19, as evidenced by the prevalence of lymphocytopenia and eosinopenia [47]. The dynamics of leukocyte populations, such as neutrophils, lymphocytes, and monocytes, were utilized to calculate immunocompetence; in contrast to non-survivors, survivors exhibited oscillatory patterns in their complete blood cell count (CBC) data [48]. However, considering the sex variations in immunological response, men were found to have a considerably greater Kyn/Trp ratio than women. Individuals with substantial lymphopenia (low lymphocyte count) demonstrated the highest Kyn:Trp ratio, suggesting that the kynurenine pathway may be active in these patients in proportion to the strength of their immune response [49]. In addition, the simulation of the kynurenine pathway has been linked to the immunological dysfunction observed in severe COVID-19 cases and has also been associated with the dysregulation of mitochondrial electron transport, specifically alterations in cytochrome-dependent oxidative phosphorylation. Cytochrome activity disruption increases oxidative stress, reduces ATP synthesis, and causes immune cell overload, all of which worsen inflammatory responses [36].

Unlike healthy controls, who usually possess the majority of circulating PBMCs, participants with COVID-19 and influenza exhibited distinct inflammatory profiles characterized by declining B-cell counts and substantial decreases in CD4 and CD8 T cells [50]. Compared to controls, COVID-19 patients displayed a greater number of plasmablasts derived from B cells, which are known to release early circulating antibodies [51]. All groups showed a similar amount of activated CD4 and CD8 circulatory cells; however, the COVID-19 participants demonstrated a notably decreased number of the three main categories of circulatory monocytes: classic, intermediate, and non-classic [50]. Monocytes from COVID-19 patients continued to function even after they had recovered, and CD169^+^ expression was identified as a potential sign of infection. Park et al. (2021) revealed that the COVID-19 group had higher numbers of CD169^+^ monocytes than the recovered group, which is associated with non-classical monocytes linked to inflammation [52]. CD169^+^ monocytes were linked to the intensity and clinical events of COVID-19 [53]. The elevated levels of monocytes in patients with acute COVID-19 were associated with systemic inflammation and immunological dysfunction. Additionally, their presence was detected even in asymptomatic convalescent individuals, suggesting that immune system alterations extended beyond the acute phase of infection [54]. However, for those with high-risk conditions such as type 2 diabetes mellitus (T2D), notable decreases were observed in the frequency of classical monocytes (CD14^Hi^ CD16^−^), along with a more pronounced case of lymphocytopenia in patients with T2D, with 1.6 times fewer CD8^+^ lymphocytes in those requiring critical care [55]. The association between early innate immune responses and the counts of adaptive lymphocyte cells (CD4 and CD8 T cells and B cells) needs to be analyzed more broadly, considering sex, age, and different comorbidities.

Severe COVID-19 infection exhibits markedly altered granularity, size, and surface marker expression, as well as a severe disruption of myelopoiesis, which reduces monocytes and neutrophils to undetectable levels. Alterations in dendritic cell quantity and migration capacity have been linked to both moderate and severe COVID-19 for a minimum of seven months following infection. Kennedy et al. (2021) investigated whether the transient changes in circulating monocytes—specifically, the expression of CCR2 and CX3CR1 observed 1 to 3 months after infection—resulted from the highest levels emigrating toward inflamed or damaged tissues, given that CCL2 was linked to the recruitment of monocytes to the lungs during infection [56].

Cell therapy has emerged as a viable option for regulating the immune response by using the regenerative capacity of immune cells to treat COVID-19. By preventing cytokine storms and re-establishing immunological homeostasis, tocilizumab—an IL-6 inhibitor—enhances this strategy, especially for patients with lymphopenia [57].

The ability of many immune cell subsets, including Treg cells [58], NK cells, mesenchymal stem cells, and T cells, to protect COVID-19 patients from serious adverse consequences has been investigated. Macrophages M1 (classically activated phenotype macrophages) and M2 (alternatively activated phenotype macrophages) regulate immune activities and are essential for COVID-19 protection and pathogenesis [50,59]. NK cells were previously shown to exhibit unique dynamics during recovery from COVID-19, strongly indicating that these cells modulate the initial immune response.

The immunological response in young people has been a focal point for investigation, revealing a distinct signature expressed by neutrophils in infected children. This signature includes a decreased expression of adhesion molecules that are crucial for neutrophil migration, an increased expression of inhibitory receptors such as leukocyte-associated immunoglobulin-like receptor 1 (LAIR-1) and programmed death ligand 1 (PD-L1), and inflammatory markers such as CD64, HLA-DR, and PECAM-1. Children with COVID-19, whether symptomatic or asymptomatic, could be distinguished by IgG antibody levels against the SARS-CoV-2 spike protein and by CD64 expression on neutrophils. Further research is needed to better understand the immunological response in children and their immune system’s reaction to infection, as insufficient attention has been given to investigating these aspects [6].

Thus, regardless of age, SARS-CoV-2 impacts the immune response at the cytokine and immune cell levels. The different mechanisms, as well as the repercussions of SARS-CoV-2-induced modifications, require further research. This review focuses mainly on cytokines and NK cells involved in innate immunity in adults.

#### 3.2.3. Role of NK Cells in the Innate Immune Response

During the initial stage of the immune response, monocytes, macrophages, dendritic cells, and innate lymphoid cells (ILCs) (NK, ILC1, ILC2, ILC3, and LTi cells) respond to molecular patterns of pathogens by secreting interferons and proinflammatory cytokines. NK cells are crucial for the immune response, as they possess receptors that transmit activation signals, as well as inhibitory receptors that regulate these responses. NK cells induce direct lysis of virus-infected cells by producing perforin and granzyme B [60], facilitating the production of inflammatory cytokines, antibody-dependent cell cytotoxicity, and interactions with immune cells such as monocytes [31].

A significant decrease in the absolute number of NK cells in peripheral blood was observed in patients with severe COVID-19 and those in intensive care units (ICUs) [4,61,62]. A shift in the distribution of NK cell subtypes and their surface markers has been identified in patients with COVID-19. Furthermore, a possible connection between an increase in the number of CD56+ CD16+ NK cells and disease severity has been suggested [14]. Severe COVID-19 results in a decrease in TNF type I response, overactivation of neutrophils and macrophages, inflammatory cytokine production, and decreased NK cells.

Both age and SARS-CoV-2 infection alter NK cell activity. The age-related processes are important in SARS-CoV-2 infection in the early stages of viral infection. NK cells are normally activated by cytokines such as type I interferons, IL-12, IL-18, and IL-15 to kill virus-infected cells and activate their capacity to produce antiviral cytokines [14]. However, in elderly patients, NK cells display a reduced ability to produce IFN-gamma in response to IL-2 stimulation [14,15]. When comparing severe COVID-19 patients in adults and the elderly, it was discovered that the former exhibited a diversion toward phenotypes (CD56low, CD16high, and CD56neg) with activation markers and inhibitory receptors, whereas the latter exhibited fewer total NK cells. The ability of both groups’ NK cells to degranulate was impaired. However, TGF-β only had an impact on the IFN-γ production of adults. This was linked to shorter hospital stays in adults, indicating that TGF-β may be able to stop systemic inflammation and excessive NK cell activation. These results demonstrated that NK cells play an age-dependent role in the development of SARS-CoV-2 infection [14,63]. In this context, NK cells are activated, and their capacity to produce antiviral IP-10 is significantly increased by IL-18, type I interferons, IL-12, IL-18, and IL-15 [64]. In older individuals with COVID-19, phenotypic changes were noted, including increased expression of CD57 and CD69 [64], indicators of overactivation, as well as up-regulation of PD-1 on NK cells [65]. However, the absolute number and subset distribution of NK cells were not linked to age. In severe COVID-19, a higher proportion of NK cells with an adaptive phenotype (NKG2C+ FcRγ -/low) correlates with elevated type I interferons, particularly in older individuals. This elevation in interferon levels is further influenced by the S1 and S2 proteins of SARS-CoV-2, which enhance the migration and secretion of IFN-γ by NK cells, indicating direct interactions with the virus. Stress signals guide NK cells to infected or inflamed tissues through chemotaxis and adhesion molecule interactions. In viral infections or persistent inflammation, the CD56bright NK subpopulation preferentially homes to these sites via chemokine receptors (CXCR3, CCR5, and CXCR6), which detect localized inflammatory cues [14]. However, transfection of lung epithelial cells with the S1 protein results in reduced degranulation and secretion of IFN by NK cells [19], accompanied by increased expression of HLA-E and NKG2A [66], which can lead to NK cell exhaustion as observed in other viral infections. NK cells’ increased expression of NKG2A during long-term hepatitis C virus (HCV) infections is linked to a compromised immune response, as HLA-E and NKG2A interactions inhibit the activity and viral clearance of NK cells. Together with decreased levels of TNF-α, granzyme B, and IFN-γ, cell markers such as NKG2A in NK cells may indicate a mechanism that both exhausts NK cells in COVID-19 patients and prevents their production [14,67]. In addition to contributing to the depletion of COVID-19 patients, the overexpression of the inhibitory receptor NKG2A on NK cells highlights a possible therapeutic target [4]. NKG2A blockade could improve antiviral immunity in chronic or long COVID infections, such as chronic HCV, as NKG2A inhibitory signaling decreases, enhancing NK cell cytotoxicity and IFN production, and increasing the CD8+ T-cell antiviral response [68,69]. However, another study using integrative systems showed that NKG2A-biased immune responses (NKG2A in NK cells and CD8 T cells) in viral infections could also play a beneficial role, as it is associated with a reduced acute and post-acute COVID-19 risk, less inflammation, and increased protective humoral immunity [68,69]. This suggests that, depending on the moment of the infection (acute, post-acute, and long COVID) and other factors, NKG2A could be a protective or detrimental factor; thus, therapeutic approaches could vary.

These modifications are demonstrated by an elevation in CD3- CD56dim CD16- NK cells and an increase in PD-1+ NK cells, indicating that these changes may play a role in the progression of severe COVID-19 cases [70]. According to a recent study, children with symptomatic but non-severe COVID-19 exhibited higher NK cell reactivity to K562 cells compared to adults with mild or severe disease or toddlers without symptoms. This suggests that infants, who are often less susceptible to severe COVID-19, may not have this deficiency. These data should be further examined, including children with severe illness, to determine whether robust NK cell activity in children may play a protective role against COVID-19 [51,65]. Moreover, the use of specific markers such as CD163 and CD25 revealed another age-related difference in NK cell response, observed not only between adults and the elderly. The use of these markers showed that elevated CD25 and a pronounced increase in CD163 are characteristic of MIS-C (multisystem inflammatory syndrome in children) compared to other groups. In one MIS-C cohort, patients exhibited reduced numbers of CD4+ and CD8+ T lymphocytes and NK cells, whereas B lymphocytes were reduced less consistently [71]. MIS-C demonstrated that SARS-CoV-2-induced endothelial dysfunction is a significant factor in the pathophysiology of shock and multi-organ failure in pediatric patients, in addition to the endothelial damage observed in adults with severe COVID-19 [72].

These findings indicate distinct differences in immune profiles between MIS-C and severe COVID-19, which may have implications for diagnosis and treatment, particularly concerning NK cell function and exhaustion [69]. The relationship between markers such as NKG2A expression and cytokine levels offers potential insights into NK cell exhaustion [73,74], paving the way for adjunct therapies to boost immune resilience. Understanding these differences is crucial for tailoring therapeutic strategies to improve outcomes in affected individuals, increasing the antiviral response without inducing an inflammatory response, which could increase the risk of severity.

Numerous viruses, including SARS-CoV-2, have developed mechanisms to evade NK cells and disrupt the host immune response [2,4] (Table 2). SARS-CoV-2 employs accessory proteins that modify host cell functions, activating inflammasomes and NF-κB pathways, which can lead to cytokine storms and cell death. Key proteins such as ORF3a and ORF7a increase inflammation and inhibit type-I interferon, which is crucial for antiviral defense [75]. ORF3a is a viroporin that inhibits global protein trafficking, including MHC-I, reducing antigens in CD8+ T cells; ORF7a mimics beta-2 microglobulin and interferes with MHC-I assembly [76]. ORF3a promotes inflammation and apoptosis in immune cells, suggesting that targeting ORF3a could enhance the host’s immune response and reduce the severe effects of COVID-19 [77].

Additionally, viroporins activate necroptosis and pyroptosis, contributing to inflammation associated with COVID-19. Research indicates that SARS-CoV-2 accessory proteins not only block interferon signaling but also aid in virion assembly and viral escape [75]. Non-structural proteins (NSPs) play a role in immune evasion by disrupting immune signaling pathways [78,79]. Ongoing research aims to develop treatments targeting these evasion strategies to enhance antiviral responses.

#### 3.2.4. Immune Response to SARS-CoV-2 in Pregnant Women

The immune system undergoes significant adaptations during pregnancy to maintain immune tolerance toward the fetus while also providing protection against pathogens. These changes are particularly pronounced in the third trimester, where alterations in humoral responses, the suppression of T-cell-mediated immunity, and shifts in immune cell composition and activity are observed [80]. Thus, the immune response induced by SARS-CoV-2 infection in pregnant women and its impact on disease severity [81], as well as on pregnancy outcomes, has been an important focus of SARS-CoV-2 research, given that the implications of this emergent virus infection in this population have not always aligned with typical expectations for a respiratory virus.

Pregnant women exhibit activated monocytes and an enhanced inflammatory response from monocytes and plasmacytoid dendritic cells (pDCs) when exposed to pathogens such as the Influenza A virus [82]. Similarly, T cells and NK cells demonstrate an increased production of IFN-γ and improved functional responses upon H1N1 virus exposure. However, the relationship between immune responses to SARS-CoV-2 infection and pregnancy is complex and not fully understood. Studies indicate that pregnant women with COVID-19, particularly in their third trimester, experience a dysregulation of innate immunity, characterized by lower levels of IFNγ and reduced expression of CD14 and HLA-DR on monocytes. This is associated with decreased levels of anti-SARS-CoV-2 IgG. This dysregulation correlates with increased risks of obstetric and perinatal complications such as preterm birth and neonatal cerebral ischemia [83]. Additionally, COVID-19 is linked to a proinflammatory shift in pregnant individuals, characterized by a reduction in Th2 cells and a lowered Treg/Th17 ratio, alongside an elevated Th1/Th2 ratio and IL-1β and IL-18 levels. These changes may lead to endothelial activation and hypertension, which are associated with adverse pregnancy outcomes [84].

Vaccination studies revealed that pregnant women respond to mRNA-based SARS-CoV-2 vaccines with coordinated Spike-specific CD4+ and CD8+ T-cell responses and T-cell memory development. However, humoral and cellular immune responses are blunted compared to women who are not pregnant [85]. This blunted response underscores the need for optimized vaccination strategies to ensure both maternal and neonatal protection. In pregnant women, mRNA vaccines produce a stronger immune response than viral vector vaccines. While both vaccines are safe and do not increase the risk of gestational complications, women receiving the mRNA vaccines exhibit high IgG titers, effective placental transfer (70–90%), and a long-lasting cellular response (≥6 months), in addition to neutralizing better variants like Omicron. The antibody levels in the umbilical cord are 1.2–1.5 times lower than those in the mother vaccinated with the mRNA vaccine, reflecting an improvement compared to being 2 times lower with the viral vector [85,86].

Overall, these findings highlight the unique immune challenges and adaptations during pregnancy related to SARS-CoV-2 infection and vaccination. Further studies are needed to determine the role of innate immunity in these responses.

## 4. Discussion

Although the number of COVID-19 cases has decreased since the pandemic began, many concerns still need to be addressed, and the literature on the severity of COVID-19 and the role of immune responses is extensive. To provide insights into potential treatments and the processes underlying the course of the disease, this review highlights the significance of ongoing research examining the relationship between SARS-CoV-2 and the immune system, focusing on the innate immune responses of cytokines and NK cells (Figure 1). Although the innate immune responses of coronavirus infections, such as SARS-CoV, MERS-CoV, and SARS-CoV-2, share commonalities, they differ in ways that explain variances in clinical outcomes.

Cytokine storms, characterized by an excessive release of IL-6, IL-1β, and TNF-alpha, remain a central determinant of disease severity in COVID-19. This hyperinflammatory state disrupts immune homeostasis, contributing to multi-organ damage. Therapeutic approaches that regulate cytokine production or block the functions of proinflammatory cytokines once they are already produced are promising strategies to mitigate disease progression.

The ACE2 receptor, highly expressed in epithelial cells of the respiratory tract, facilitates viral entry and activates inflammatory pathways. However, the full extent of endothelial damage caused by SARS-CoV-2 requires further exploration.

SARS-CoV-2’s ability to evade immune detection by disrupting interferon production and other key cytokines highlights the urgency of developing therapies to restore initial immune function and control excessive inflammation. Restoring interferon signaling may enhance the immune response and reduce disease severity, offering a novel direction for therapeutic research. Furthermore, limited explanations regarding the activation of the NLRP3 inflammasome and the role of viral accessory proteins in immune evasion underscore the need for deeper investigation.

Natural Killer cells are vital to the antiviral immune response, as they directly attack infected cells and secrete cytokines that regulate the broader immune system. However, in COVID-19, their function becomes impaired, with reductions in cell counts, diminished degranulation capacity, reduced IFN-γ production, and increased expression of exhaustion markers such as NKG2A. Elevated TGF-β levels and the overexpression of inhibitory receptors exacerbate systemic inflammation, further complicating disease management. Age-related changes in innate immunity significantly impact NK cell activity. Older patients with severe COVID-19 show altered NK cell phenotypes (CD56lowCD16high) and increased expression of inhibitory markers such as PD-1. These observations not only underscore the need for tailored therapeutic strategies to enhance NK cell function in vulnerable populations through cytokine response modulation but also for specifically blocking exhaustion markers/inhibitory receptors such as NKG2A and PD-1. The interplay between NKG2A expression and cytokine levels offers potential insights into NK cell exhaustion and immunomodulation, paving the way for adjunct therapies to boost immune resilience.

COVID-19’s dysregulated immune responses are similar to sepsis, trauma, and severe burns, ultimately leading to systemic inflammation and multi-organ failure. Exploring these shared mechanisms may uncover broader strategies for managing critical illnesses.

This review presents several strengths, such as integrating studies addressing the innate immune response in COVID-19, providing a comprehensive overview of the interaction between the virus and the immune system. It highlights the importance of NK cells and cytokine profiles in disease severity and their effects in the adaptive immune response and protection, which may guide future therapeutic strategies. Understanding the virus’s interaction with its receptors in different cells and organs, along with innate immune components—especially the initial innate immune responses in infected cells—may yield insights into its pathogenesis by identifying innate immune cells such as NK cells, dendritic cells, neutrophils, and macrophages (Figure 1). Identifying inflammatory biomarkers and cytokines across different stages of infection may provide valuable predictive tools for disease severity and treatment response.

However, this review also has limitations in its methodology, such as the exclusive focus on articles in English, which may exclude relevant research in other languages and omit information about some world regions significantly impacted by SARS-CoV-2. In addition, reliance on original studies may limit the generalizability of the findings, as systematic reviews that could offer a broader context were omitted. Finally, the variability in the designs of the included studies may complicate direct comparisons of results. This review also has scientific limitations due to its specific focus on the innate immune responses of cytokines and NK cells, mainly in natural SARS-CoV-2 infections. Thus, a detailed description of the current knowledge on other relevant areas of COVID-19 immune responses, such as the role of different innate immune cell components (monocytes, macrophages, dendritic cells, neutrophils, etc.), the role of the adaptive immune response and memory responses, the immune responses during vaccination, the role of immune responses in long COVID, and the immune responses in specific populations, including pediatrics and pregnant women, should be the focus of additional literature review studies.

## 5. Conclusions

Understanding the complex interplay between SARS-CoV-2 and the host immune response is essential for developing effective therapeutic strategies for COVID-19. Interventions to address the unique challenges faced by vulnerable populations, particularly those with age-related factors and comorbidities, as well as pregnant women, are crucial for improving patient outcomes. Identifying inflammatory biomarkers can facilitate personalized treatment plans, while elucidating the mechanisms of immune evasion employed by the virus, including the roles of accessory proteins and the activation of the NLRP3 inflammasome, will be vital for restoring normal immune function. This research not only has implications for COVID-19 but also extends to other critical inflammatory conditions, such as sepsis and trauma, where dysregulated immune responses can lead to severe complications. Continued investigation into the immune response dynamics, particularly the interactions between cytokines and immune cell behavior, will enhance our understanding of disease progression and tissue regeneration. By focusing on these areas, we can develop adjunct therapies that strengthen the immune response, improving outcomes for patients affected by COVID-19 and other serious inflammatory diseases. The insights gained from this research have the potential to transform our approach to managing these complex health challenges, paving the way for more effective treatments and improved patient care. This review underscores the importance of investigating immune system interactions, particularly the behavior of immune cells and cytokine profiles in patients with varying age groups and comorbidities. Addressing these gaps may not only improve patient outcomes but could also enhance preparedness for future pandemics. Current evidence reinforces the need for a balanced approach to immune regulation, emphasizing that both excessive inflammation and inadequate responses can be detrimental to recovery. The pivotal role of inflammation in disease outcomes underscores the urgent need for targeted interventions that modulate inflammatory pathways and restore immune function, with implications for improving outcomes in COVID-19 and other inflammatory diseases.

## Figures and Tables

**Table 1:** Eligibility criteria for study inclusion in the first group of articles.

Eligibility Criteria	Description
Year of Publication	Articles published between 2020 and 2024.
Language	Only articles in English.
Open Access	Only articles with full-text open access and free availability.
Type of Publication	Only original research articles (excluding reviews or editorials).
Focus on Humans	Studies focused on human populations (men, women, children, and adults).
Topic Relevance	Focused on critical mechanisms and fundamental processes related to the topic.

**Table 2:** Impact of SARS-CoV-2 proteins on NK cell dysfunction and cytokine signaling.

Viral Protein	Effect on NK Cell	Impact on Cytokines	Consequence
Spike	↓ MICA/B (inhibits activation)	↑ IL-6, ↓ IFN-γ	Phenotypic exhaustion of NK cells.
ORF3a	↑ PD-1/TIM-3 (exhaustion)	↑ TGF-β (immunosuppression)	Loss of cytotoxic function of NK cells.
NSP1	↓ CD69 (activation)	Alteration of type I IFN pathways	Poor antiviral response (less immune cell activation, including NK cells).

The viral proteins affect NK cells and cytokines. NK cell exhaustion demonstrates a complex interaction between cellular defects associated with inflammation and immune system failure. (↓ denotes reduction, ↑ denotes increase)

## Data Availability

No new data were created or analyzed in this study.

## Supplementary Materials

The following supporting information can be downloaded at https://www.mdpi.com/article/10.3390/cells14110763/s1, Figure S1: General framework of the literature search and analysis used for the review; Table S1: Results of the bibliographical search after applying the inclusion and exclusion criteria; Table S2: Main results from the primary selected articles during the literature search.
