# Supplementary material for: Dynamics of Innate Immunity in SARS-CoV-2 Infections: Exploring the Impact of Natural Killer Cells, Inflammatory Responses, Viral Evasion Strategies, and Severity
Source: Cells. 2025 May 22;14(11):763. doi: 10.3390/cells14110763 (PMC12153545; doi:10.3390/cells14110763)
Supplement: Supplementary file 1 [file cells-14-00763-s001.zip › cells-3572332-supplementary.pdf]

Supplementary Information

1. Methodology of the literature review

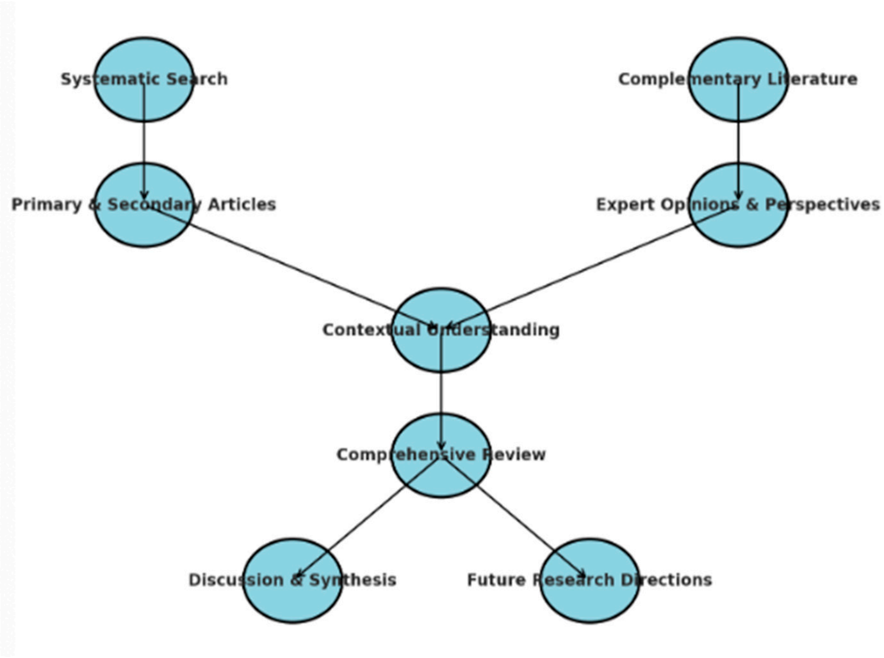

Supplementary Figure S1. General framework of literature search and analysis used for the review. This flowchart illustrates the process of integrating complementary literature into this review. Offering contextual understanding, presenting expert opinions, and exploring theoretical perspectives. The inclusion criteria were defined to focus on articles published between 2020 and 2024, ensuring that the most recent findings were considered. In addition to the primary and secondary articles, complementary literature was also reviewed to provide a broader context and enhance the understanding of the topic.

2. Results of the bibliographical search

Supplementary Table S1. Results of the bibliographical search after applying the inclusion and exclusion criteria.

Search Results: clean filtered
((((("immunity, innate"[MeSH Terms] OR ("immunity"[All Fields] AND "innate"[All Fields]) OR "innate immunity"[All Fields] OR ("innate"[All Fields] AND "immune"[All Fields] AND "responses"[All Fields]) OR "innate immune responses"[All Fields]) AND ("killer cells, natural"[MeSH Terms] OR ("killer"[All Fields] AND "cells"[All Fields] AND "natural"[All Fields]) OR "natural killer cells"[All Fields] OR ("killer"[All Fields] AND "cell"[All Fields] AND "natural"[All

Fields]) OR "killer cell natural"[All Fields]) AND ("coronavirus"[MeSH Terms] OR "coronavirus"[All Fields] OR "coronaviruses"[All Fields])) OR "SARS-COV2"[All Fields]) AND "hasabstract"[All Fields]) OR ("immunity, innate"[MeSH Terms] AND ("coronavirus"[MeSH Terms] OR "coronavirus"[All Fields] OR "coronaviruses"[All Fields]) AND "hasabstract"[All Fields])) AND ((("immunity, innate"[MeSH Terms] AND ("coronavirus"[MeSH Terms] OR "coronavirus"[All Fields] OR "coronaviruses"[All Fields])) OR "SARS-COV2"[All Fields]) AND "hasabstract"[All Fields])	
Results #1:	1969 to 2024= 6585
Selection Criteria	Free full text, 5 years, humans, English, female, male, Child: birth -18 yrs, Adult: 19 +yrs, 65 + yrs. Published between 2020 to 2024.
Exclusion Criteria	Preprint, systematic review and meta-analysis.
Results #2:	2020 to 2024 = 894
Filtered	
Total information research	44

Supplementary Table S2. Main results from the primary selected articles during the literature search.

Author	Year	Title	Population	Results
Lee PY	2020	Distinct clinical and immunological features of SARS-CoV- 2-induced multisystem inflammatory syndrome in children	Pediatrics population- symptoms moderate to severe	Common symptoms included conjunctivitis (57%), gastrointestinal issues (54%), hypotension or shock (54%), and skin rash (36%). SARS-CoV-2 infection was confirmed in all patients, with a 61% PCR positivity rate and 95% serology positivity. Lymphopenia (75%), thrombocytopenia (64%), and elevated inflammatory markers such as D-dimer (96%) and ferritin (86%) were observed. Additionally, 39% of patients exhibited cardiac dysfunction, with ventricular ejection fractions below 55%.

Fawaz Alzaid	2020	Monocytopenia, monocyte morphological anomalies and hyperinflammation characterize severe COVID-19 in type 2 diabetes	Diabetic and non- diabetic men and women	The study observed significant reductions in monocyte frequency among type 2 diabetes (T2D) patients with COVID-19, particularly a 1.4-fold decrease in classical monocytes (CD14^Hi CD16^−). Both T2D and non-diabetic patients exhibited lymphocytopenia, but it was more pronounced in T2D patients, with a 1.6-fold reduction in CD8^+ lymphocytes in those needing intensive care. T2D patients also had larger monocytes, with a 1.4-fold increase in size and a 1.8-fold higher frequency compared to non- diabetics. Inflammatory markers like IL-6, IL-8, and CCL2 were elevated in T2D patients, with increases ranging from 1.6 to 4.8 times. Factores de Riesgo Asociados La prevalencia de hipertensión y niveles elevados de hemoglobina glicosilada (HbA1c) fueron significativamente más altos en pacientes con T2D en comparación con los no diabéticos, lo que indica que estos factores pueden agravar la<br>severidad de la infección.
Luana Lionetto	2020	Increased kynurenine- to-tryptophan ratio in the serum of patients infected with SARS- CoV2: An observational cohort study	La distribución de género fue de 181 hombres y 213 mujeres en total. Además, se incluyeron 239 sujetos sanos como controles, de los cuales 87 eran hombres y 152 eran mujeres.	The Kyn:Trp ratio was positively correlated with age and severity of infection, indicating that as age and severity increase, so does the activation of the kynurenine pathway. Men were found to have a significantly higher Kyn:Trp ratio than women, suggesting sex differences in immune response. Patients with severe lymphopenia (low lymphocyte count) showed the highest levels of Kyn:Trp, suggesting that activation of the kynurenin pathway could be related to the severity of the immune response in these patients.
Thomas Sonnweber	2020	Persisting alterations of iron		Anemia was observed in 9.2% of patients, being more frequent in men (12%) than in women (5%) and 70% of anemic patients had anemia of inflammation (AI). Approximately, 38% of patients still showed hyperferritinemia, more common in those with severe or critical COVID-19. Anemic patients showed elevated levels

		homeostasis in COVID-19 are associated with non- resolving lung pathologies and poor patients' performance: a prospective observational cohort study	Male and female hospitalized patients- Mild to critical symptoms	of interleukin-6 (IL-6) and C-reactive protein (CRP). And the correlation was observed between elevated ferritin levels and cytokine expression in peripheral blood mononuclear cells (PBMCs). Patients with severe COVID-19 showed increased expression of hepcidin, a key regulator of iron homeostasis. Evaluation of serum iron parameters could be an accessible tool for monitoring disease resolution.
Ulrich C	2020	NLRP3 Inflammasome Activation in Hemodialysis and Hypertensive Patients with Intact Kidney Function	Adult patients with comorbidities.	"Quantitative Results: Body Mass Index (BMI): Hypertensive patients had a significantly higher BMI (28.8) compared to HD patients (24.5) ($p = 0.022$).<br>Caspase-1: A higher frequency of caspase-1 positive monocytes was observed in hypertensive patients ($p < 0.001$). IL-1β secretion: IL-1β levels were significantly higher in hypertensive patients compared to HD patients. Pyroptosis: The rate of pyroptosis was higher in HD patients (6.3%) compared to hypertensive patients (0.6%), although statistical significance was not reached ($p = 0.08$). Qualitative Results: Inflammatory State: Although C-reactive protein (CRP) levels did not show significant differences between the two groups, hypertensive patients presented a more active inflammatory state, reflected in higher levels of IL-6. Inflammasome Activation: It was concluded that
Morrison AR	2020	Clinical characteristics and predictors of survival in adults with coronavirus disease 2019 receiving tocilizumab	Male and female population with mild, moderate and severe symptoms.	In a study of 81 adult COVID-19 patients treated with tocilizumab, 28-day mortality was 43.2%. Early tocilizumab intervention (within 12 days) improved survival significantly, while a SOFA score ≥8 indicated higher mortality risk. Clinical response was notably higher in survivors, underscoring the importance of early treatment and

				monitoring for adverse effects and infections.
Mudd PA	2020	Distinct inflammatory profiles distinguish COVID-19 from influenza with limited contributions from cytokine storm	In the COVID-19 group, 44% of patients were women, while in the influenza group, 58% were women.	The study shows that patients with COVID-19 have a less pronounced inflammatory profile than patients with influenza, challenging the notion of a frequent cytokine storm. Although some present elevations in certain cytokines, most do not show significantly higher levels than in influenza, suggesting more selective and less broad inflammation. A pattern of immunosuppression was identified in COVID-19, with lower HLA-DR expression in mononuclear cells. In quantitative terms, the number of lymphocytes is similar between both groups, but patients with COVID-19 have fewer monocytes. Cytokines, such as IL-6 and G-CSF, although associated with a higher risk of death, are not statistically higher than in influenza. These findings imply different clinical management for COVID-19.
Chen X	2020	Characteristics of immune cells and cytokines in patients with coronavirus disease 2019 in Guangzhou, China	In the study, the COVID-19 patient population consisted of 80 patients with mild symptoms and 22 patients with severe symptoms.	T Cells: CD3+ T cells: Showed a significant decrease in severe COVID-19 patients compared to healthy controls and mild cases, persisting post-recovery. CD4+ T cells: Significantly reduced in severe cases; remained low after recovery. CD8+ T cells: Also reduced in severe cases, though their proportion was not significantly altered.<br>B Cells: Decreased in mild cases and further reduced in severe cases, with levels staying low post-recovery.<br>NK Cells: Significantly decreased in severe cases, tending to normalize after recovery.<br>Regulatory T Cells (Tregs): Increased in both mild and severe cases compared to healthy controls, persisting after recovery.<br>Cytokines: IL-6 and IL-10: Elevated in severe cases;<br>IL-6 remained high post-recovery, whereas IL-10 normalized.

Zheng Y	2020	A human circulating immune cell landscape in aging and COVID-19	The study analyzed different population cohorts, which included both young adults and older adults, as well as patients with COVID- 19 at different stages of the disease.	The study explores how aging and COVID-19 infection affect immune cells. First, it shows that aging shifts T cells from naïve and memory types to effector, cytotoxic, exhausted, and regulatory forms. This change is accompanied by increases in late-stage NK cells, age-related B cells, inflammatory monocytes, and dysfunctional dendritic cells. Moreover, certain cell subtypes express more genes related to coronavirus susceptibility as people age. Furthermore, COVID- 19 infection worsens these age-related immune changes by increasing the expression of genes linked to inflammation and cellular aging. The study also observed a decrease in the diversity of TCR and BCR, with more clonal expansion in CD8+ effector T cells and age-associated B cells. Aging also alters chromatin accessibility, boosting the activity of AP-1 transcription factors in immune cells.<br>In terms of numbers, the study finds a 10-15% decrease in T and B cells in older adults compared to younger ones, along with a 7-10% increase in monocytes. Clinically, older COVID-19 patients have more inflammatory monocytes and fewer T cells, leading to worse outcomes and slower recovery compared to younger patients. In conclusion, the study highlights the significant impact of aging and COVID-19 on immune cell composition and function, emphasizing their role in disease susceptibility and response in the elderly.
Masselli E	2020	NK cells: A double edge sword against SARS-CoV-2	Specific information about the study population's gender and age is not provided, but variations in the number and function of NK cells during SARS-CoV-2 infection are observed to correlate with the severity of clinical	The T cells, CD8+ T cells, and NK cells are significantly reduced. This reduction is linked to increased expression of inhibitory receptors like NKG2A, indicating NK cell exhaustion and compromised immune function. The role of cytokines, such as IL-6 and IL-10, which may inhibit NK cell activity, contributing to severe complications like multiorgan

			presentation and outcomes in COVID- 19 patients.	failure. The research also examines the recruitment of NK cells to the lungs, as seen in bronchoalveolar lavage fluid samples from COVID-19 patients. The genetic and molecular aspects of immune responses, with studies on gene expression changes due to aging and COVID-19. It also covers the development of vaccines and their role in combating the pandemic, alongside clinical trials exploring treatments like probiotics for symptomatic relief and viral clearance.
Elyse Latreille	2021	Interactions of Influenza and SARS- CoV-2 with the Lung Endothelium: Similarities, Differences, and Implications for Therapy	Hospitalized adult patients-symptoms moderate to severe	In post-mortem studies, microthrombi in pulmonary vessels were found to be nine times more prevalent in patients with COVID-19 compared to those with influenza. In critically ill COVID-19 patients, IL-6 levels were nearly 10 times higher compared to less severe patients, suggesting that IL-6 could be a marker of poor prognosis. Patients with severe COVID-19 showed elevated levels of D-dimer, with a significant increase in the incidence of disseminated intravascular coagulation (DIC).
Young BE	2021	Antibody responses after a single dose of ChAdOx1 nCoV-19 vaccine in healthcare workers previously infected with SARS-CoV-2	Predominant Woman population-symptoms moderate to severe	The context provides a summary of various studies on COVID-19, highlighting key findings such as the robust immune response from single-dose ChAdOx1 nCoV-19 vaccines in previously infected individuals, the role of vitamin C and probiotics in treatment, and the impact of dexamethasone on immune modulation. It also discusses the variability in immune responses, the effectiveness of mRNA vaccines, and the potential link between SARS-CoV-2 vaccines and myocarditis.
Camille Lenoir	2021	Impact of SARS-CoV-2 Infection (COVID-19) on Cytochromes P450 Activity Assessed by the Geneva Cocktail	81% of the population corresponds to the male population with moderate to severe disease	The results of the study indicate that SARS-CoV-2 infection significantly affects the activity of drug- metabolizing enzymes, specifically cytochrome P450 (CYP). A decrease in CYP1A2, CYP2C19, and CYP3A activities was observed, while CYP2B6 and CYP2C9 activities increased. These changes are correlated with

				elevated levels of inflammatory markers such as CRP and IL-6.
Allison E. Kennedy	2021	Lasting Changes to Circulating Leukocytes in People with Mild SARS-CoV- 2 Infections	Adult Men and Women-Mild Symptoms	Individuals recovering from mild COVID-19 show prolonged immune activation, with distinct T cell and monocyte changes not seen in other respiratory infections. This includes heightened T cell activation and systemic inflammation persisting 1-3 months post-infection.
Ge Chen	2021	Differential immune responses in pregnant patients recovered from COVID-19	Pregnant woman	The study explored how pregnant women recovering from COVID-19 respond immunologically, using advanced techniques like single-cell RNA sequencing. It found that despite a general reduction in lymphocytes, these women had heightened activation in certain immune cells like NK, NKT, and MAIT, indicating a robust antiviral response. Clinically, pregnant women showed higher levels of inflammation markers such as neutrophils, hs-CRP, and IL-6 compared to non- pregnant women. Their immune cells, particularly monocytes, responded more strongly to interferons, and T cell responses remained functional, suggesting that their immune systems, while altered, are still capable of effectively fighting the virus.
Michael Z. Zulu	2021	Obesity Correlates With Pronounced Aberrant Innate Immune Responses in Hospitalized Aged COVID-19 Patients	The study focuses on the population of patients hospitalized with COVID-19, specifically in two age groups: young (under 60 years of age) and the elderly (over 60 years of age). In total, 39 young patients and 48 elderly patients were analyzed, classified according to their body mass index (BMI) into three categories: thin (BMI ≤ 24.9 kg/m²), overweight (25-29.9 kg/m²) and obese (≥30 kg/m²).	A significant negative correlation was observed between body mass index (BMI) and certain subsets of immune cells, such as dendritic cells and monocytes, especially in older patients. This suggests that obesity exacerbates immune dysfunction, affecting the immune system's ability to respond appropriately to SARS-CoV-2 infection. In addition, an increase in the production of proinflammatory cytokines, such as TNF-α and IL- 6, was identified in response to stimulation by bacterial agonists, indicating an alteration in the inflammatory response. The expression of activation and maturation markers in monocytes and dendritic cells was also

				affected, suggesting a compromise in the function of these cells in the immune response.
Seery V	2021	Blood neutrophils from children with COVID-19 exhibit both inflammatory and anti-inflammatory markers	Children with mild symptoms	The study involved 203 children, including 182 with COVID-19, 21 with MIS-C, and 40 healthy controls. Most COVID-19 cases in children were mild. Neutrophils in COVID-19 children showed altered marker expression, with reduced CD11b, CD66b, and L-selectin, but increased HLA-DR, CD64, PECAM-1, LAIR-1, and PD-L1 compared to controls, suggesting a less tissue-invasive profile which might protect lung function. CD64 and IgG antibody levels were higher in symptomatic children. No significant differences in cytokine production or NET release were observed between COVID-19 children and healthy controls. In the MIS-C group, 90% tested positive for IgG antibodies.
Kuśnierz-Cabala	2021	Diagnostic Significance of Serum Galectin-3 in Hospitalized Patients with COVID-19—A Preliminary Study	Hospitalized Patients	Galectin-3 levels: Serum galectin-3 was significantly higher in patients who developed pneumonia (13.30 ng/mL) compared to those without pneumonia (8.55 ng/mL). Patients who required ICU showed even higher levels (23.46 ng/mL). Significant positive correlations were found between galectin-3 and several inflammatory markers (IL-6, CRP, ferritin, PTX-3) and a marker of endothelial injury (sFlt-1). Galectin-3 also showed correlations with length of hospital stay. Galectin-3 showed a sensitivity of 52% and a specificity of 86% for the diagnosis of COVID-19 pneumonia, and a sensitivity of 78% and specificity of 90% for the need for treatment in the ICU. The correlation between galectin-3 and sFlt-1 suggests that endothelial dysfunction could be an important factor in the pathology of COVID-19, which could have implications for the clinical management of patients.
Borgel D	2021	Endothelial Dysfunction as a	Patients under 18 years of age diagnosed with SARS-	Significant correlations were found between endothelial marker levels and

		Component of Severe Acute Respiratory Syndrome Coronavirus 2-Related Multisystem Inflammatory Syndrome in Children with Shock	CoV-2- related multisystem inflammatory syndrome (MIS-C) and shock	shock severity (SIV): Angiopoietin-2: r = 0.45, p = 0.016. sE-selectin: r = 0.53, p = 0.04. von Willebrand factor: r = 0.46, p = 0.013. Angiopoietin-2/angiopoietin-1 ratio: r = 0.46, p = 0.012. Clinical manifestations: 93% of patients had acute heart failure. Most patients (89%) required hemodynamic support with inotropics and/or vasopressors. It is suggested that endothelial dysfunction is associated with systemic hyperinflammation and may be an underlying mechanism in the development of shock in patients with MIS-C. This suggests that hyperinflammation may contribute to endothelial dysfunction and, therefore, to the severe cardiovascular manifestations observed in these patients.
Chen G	2021	Differential immune responses in pregnant patients recovered from COVID-19	Pregnant woman and not pregnant woman	Pregnant women with COVID-19 show a distinct clinical profile, including increased lymphopenia and higher levels of inflammation markers (hs-CRP and IL-6) compared to non-pregnant women. They demonstrate stronger immune responses, like activated NK, NKT, and MAIT cells, and heightened responses to interferons. Quantitatively, they have lower lymphocyte and higher neutrophil counts, with IL-6 and hs-CRP levels substantially elevated. Single-cell RNA sequencing indicates a higher proportion of myeloid cells and fewer cytotoxic CD8 T cells in pregnant women.
Liu S	2021	Effects of Neutrophil- to-Lymphocyte Ratio Combined With Interleukin-6 in Predicting 28-Day Mortality in Patients With Sepsis	Adult patients with moderate to severe symptoms	Interleukin-6 (IL-6) showed the strongest correlation with severity scores (APACHE II and SOFA) in sepsis patients, followed by the neutrophil-lymphocyte ratio (NLR) and procalcitonin (PCT). NLR and IL-6 were identified as independent predictors of 28-day mortality, useful for risk stratification and guiding interventions. ROC curve analysis revealed that combining NLR and IL-6 (AUC 0.904) offers excellent mortality prediction. The Cox model showed significant odds ratios for NLR (1.281) and IL-6 (1.017),

				highlighting their potential in improving mortality prediction and clinical management.
Della-Torre E	2021	Respiratory Impairment Predicts Response to IL-1 and IL-6 Blockade in COVID-19 Patients With Severe Pneumonia and Hyper-Inflammation	Mostly men-severe COVID-19	The findings suggest that IL-1 and IL-6 blocking therapies are most effective when initiated in the early stages of the disease, before severe respiratory failure sets in.The results underscore the need to identify clinical and serological variables that can predict response to anti-cytokine therapies, which could optimize the management of patients with severe COVID-19.
Ling L	2021	Longitudinal Cytokine Profile in Patients With Mild to Critical COVID-19.	75% of men with mild intensity, 26.7% with moderate intensity, and 65% with severe intensity	The study identified 22 cytokines, including IL-6, IL-8, IL-10, IL-18, and IP-10, that correlate with COVID-19 severity, showing increased levels with disease progression. Eleven cytokines exhibited phase differences, with seven increasing and four decreasing in severe cases. IL-8, IP-10, and MDC emerged as key early biomarkers for predicting severity, while MCP-1 was linked to critical care metrics. These findings emphasize the importance of cytokine profiling and immunomodulation in managing severe COVID-19.
Zhang Q	2021	Inflammation and Antiviral Immune Response Associated With Severe Progression of COVID-19	67% men and 33% with severe symptoms, 65% men and 95% women with mild symptoms, 58% men and 42% women healthy controls. Additionally, 22 patients with Influenza A (H1N1) were admitted for additional comparisons.	Overactivation of myeloid cells (monocytes and neutrophils) and poor function of T cells were observed, suggesting an imbalance in the immune response. Gene Regulation: Four key genes (TLR6, MMP9, SKAP1, and LAG3) were identified that showed significantly altered expression patterns in severe patients compared to those with mild disease and healthy controls. TLR6 and MMP9 were associated with neutrophil-mediated inflammatory response, while SKAP1 and LAG3 were related to T-cell function. Alterations in Interferon Response: Severe SARS-CoV-2 infection was observed to largely silence the response of type I interferons, which could contribute to the hypercytokinemia observed in these patients.

Moin ASM	2021	Identification of macrophage activation-related biomarkers in obese type 2 diabetes that may be indicative of enhanced respiratory risk in COVID-19	Population with type 2 DM = 23 and controls without type 2 DM = 23	M1 and M2 Macrophage Activation: Subjects with obese type 2 diabetes (OT2D) showed elevated activation markers for both M1 (e.g., CD80, CD38) and M2 macrophages (e.g., TGF-β1, CD163, MMP7, MMP9) compared to controls, indicating a chronic inflammatory state. Cytokine Levels: In OT2D, increased levels of cytokines/chemokines like CXCL1 and CXCL5 were present, while CXCL9 and CXCL10 were lower. Glucose Normalization: Acute glucose normalization did not affect macrophage activation in OT2D, suggesting persistent inflammation independent of glycemic control. Clinical Implications: Persistent macrophage activation in OT2D may increase susceptibility to infections like ARDS, underscoring the need for treatments targeting chronic inflammation in this population.
Petrey AC	2021	Cytokine release syndrome in COVID- 19: Innate immune, vascular, and platelet pathogenic factors differ in severity of disease and sex	Population studied: 22 hospitalized, 13 men and 9 women classified according to severity (14 with moderate infection-non-ICU and 8 with severe infection-ICU)	The study on cytokine release syndrome in COVID- 19 patients revealed that patients had elevated levels of pro-inflammatory cytokines (IL-6, IL-8, TNF-α) and vascular remodeling factors (PDGF- AA, sCD40L), along with anti-inflammatory cytokines (IL-10, IL-1RA). Severe patients showed higher SOFA scores and differences in immune response between sexes, with men having higher levels of IL-4 and sCD40L. These biomarkers are crucial for assessing disease severity and predicting severe complications such as acute respiratory distress syndrome (ARDS)
Zafer Yildirim	2021	Genetic and epigenetic factors associated with increased severity of Covid-19	The context provided suggests that although SARS-CoV-2 infection rates are similar between men and women, disease severity and mortality rate are significantly higher in men. Men have a 1.7 times	Genetic variants are identified in genes such as ACE2 and TMPRSS2, which influence the susceptibility and severity of the disease. Variants in immune system genes, such as interferons, are also linked to critical cases. Likewise, specific loci are found, such as 3p21.31 and 9q34.2, related to the severity of COVID-19, affecting the

			higher risk of developing severe forms of COVID-19 compared to women.	expression of genes that regulate the immune and inflammatory response. Epigenetic mechanisms, such as DNA methylation, also play a regulatory role. Gender differences in severity are attributed to hormonal factors and X chromosome inactivation, which could influence the expression of genes such as ACE2.
Casado JL	2021	Expansion of CD56dimCD16neg NK Cell Subset and Increased Inhibitory KIRs in Hospitalized COVID-19 Patients	Population of hospitalized male and female patients, with moderate symptoms of COVID-19	The study included 80 participants: 20 hospitalized COVID-19 patients, 30 convalescents, and 30 uninfected individuals. Hospitalized patients were older, predominantly male, with higher BMI and more comorbidities. The study found a reduction in total NK cells among COVID-19 patients compared to uninfected individuals, with an expansion of the CD56^dim CD16^neg subset and depletion of CD56^dim CD16^dim and CD56^dim CD16^bright subsets. Additionally, there was increased expression of inhibitory receptors (KIR2DL1/S1) in COVID-19 patients. In COVID-19 patients exhibited an altered NK cell profile, with expansions of less cytotoxic subsets and elevated pro-inflammatory cytokines like IFN-γ and IL-6 linked to specific KIR receptors. This alteration suggests a compromised innate immune defense against SARS-CoV-2. The study implies that targeting cytokines like IL-6 in therapies could enhance antiviral immunity in severe COVID-19 cases. In summary, significant disruptions in NK cell distribution and function in hospitalized COVID-19 patients may affect immune responses and inform treatment strategies.
Cunningha m L	2021	Perforin, COVID-19 and a possible pathogenic auto-inflammatory feedback loop	Gender: Women are observed to have higher levels of perforin expression compared to men, which could contribute to lower COVID-19 mortality in women.	Perforin levels decrease with age and are lower in men, potentially explaining higher COVID-19 mortality in older adults and men. Women have higher perforin levels. Obesity and conditions like cardiovascular disease and diabetes reduce perforin levels and NK cell activity, increasing severe COVID-19 risk.

				Reduced perforin and NK cell dysfunction can cause excessive cytokine production, particularly IL-6, which is linked to higher COVID-19 mortality. Mutations in the perforin gene may increase COVID-19 mortality risk, indicating perforin's role in disease severity.
Benjamin Kramer	2021	Early IFN-a signatures and persistent dysfunction are distinguishing features of NK cells in severe COVID-19	Although the study included both men and women, specific gender distribution details are not provided in the shared data. Patients were classified according to the severity of the disease using the World Health Organization (WHO) ordinal scale,	NK cells play a crucial role, and their dysfunction in severe cases, with a reduction in the production of key cytokines such as IFN-γ and TNF-α, can contribute to disease severity and complications such as pulmonary fibrosis. In patients with post- acute sequelae of COVID-19, unique dynamics of SARS-CoV-2 and CMV-specific CD8(+) T cells are observed during recovery, suggesting that these responses are critical for recovery and may influence long-term outcomes. Type I interferon activity is impaired in severe cases, and elevated levels of IFN-α in early stages correlate with increased expression of interferon-stimulated genes in NK cells. In addition, inflammatory markers such as CRP, IL-6, and TNF-α show significant elevation, reflecting the intense inflammatory response. Probiotic supplementation has been shown to increase SARS-CoV-2-specific IgM and IgG, suggesting a potential role in modulating the immune response, although no significant changes in fecal microbiota composition were observed.
Bao C	2021	Natural killer cells associated with SARS- CoV-2 viral RNA shedding, antibody response and mortality in COVID-19 patients	Adult population, but not to refer to the distinction by gender group. In patients with severe and non-severe symptoms.	The study by Bao et al. (2021) examined 168 COVID-19 patients, categorized into severe and non-severe groups, to investigate the relationship between NK cells, viral RNA clearance, antibody response, and mortality. Severe patients showed significantly lower counts of NK cells and other lymphocytes. Higher counts of T cells and NK cells correlated with faster viral RNA clearance and quicker antibody response. Lymphocytopenia was associated with

				poorer survival and prolonged viral clearance, particularly in cases with low NK cell counts. The findings highlight the critical role of NK cells in the immune response against SARS- CoV-2, suggesting their potential as a severity marker and therapeutic target. NK cell therapy could be an effective treatment for COVID-19 without the risk of graft-versus-host disease.
Dong G	2022	Hemophagocytosis, hyper-inflammatory responses, and multiple organ damages in COVID- 19-associated hyperferritinemia	65.28% of men had hyperferritinemia and 38.7% without hyperferritinemia.	Patients with hyperferritinemia showed higher rates of fever, thrombocytopenia, ARDS, and AKI, alongside more common hypertension and diabetes, compared to those without hyperferritinemia. Elevated levels of IL-6, D-dimer, and hsCRP were also significant in this group. In- hospital mortality was notably higher at 37.50% for hyperferritinemia patients versus 4.03% for others. Kaplan-Meier analysis confirmed hyperferritinemia as a significant predictor of poor COVID-19 prognosis.
Milad Zandi	2022	The role of SARS-CoV- 2 accessory proteins in immune evasion	Population: A particular population group is not specified in the text. However, it is mentioned that children have durable immune responses against certain SARS- CoV-2 accessory proteins, suggesting possible variability in the immune response according to age.	The study highlights that SARS-CoV-2 accessory proteins like ORF3a, ORF6, ORF7a, ORF7b, ORF8, and ORF9b act as interferon antagonists, impeding immune responses by interfering with signaling pathways such as STAT1 and STAT2 phosphorylation. These proteins facilitate immune evasion by affecting key cellular processes, including inflammasome activation, apoptosis, and IFN synthesis. Quantitatively, these proteins can suppress over 40% of ISRE promoter activity, crucial for antiviral defense. Plasma from recovered COVID-19 patients contains antibodies against these proteins, highlighting an adaptive immune response. These findings underscore their role in immune evasion and virus pathogenesis, making them promising targets for antiviral therapies.

Begum Cosar	2022	SARS-CoV-2 Mutations and their Viral Variants	Although the text does not explicitly mention gender in relation to SARS-CoV-2 infection, previous studies have indicated that there are differences in susceptibility and severity of the disease between genders, with some reports suggesting that men may be at increased risk of developing severe forms of COVID-19.	SARS-CoV-2 has accumulated thousands of mutations, many of which do not significantly affect its spread or virulence. However, some mutations in the spike protein, particularly in the receptor-binding domain (RBD), can increase the severity of infection and allow the virus to evade immune protection. These mutations are crucial for the development of vaccines and antiviral treatments, as variants like B.1.1.7, B.1.351, and P.1 have shown increased transmission capacity and, in some cases, resistance to neutralization by vaccine- generated antibodies. The virus's high mutation rate facilitates its rapid evolution, posing challenges for developing effective therapeutic strategies. Approximately 4000 mutations have been documented in the S protein gene, with 89 located in the RBD, representing 58% of all mutations in this region. Variants like D614G have reached significant prevalence, up to 56% in certain populations, while others like L452R and E484K have been identified in a high percentage of samples in specific regions, indicating their rapid spread. The B.1.1.7 variant is associated with a 30% increase in transmissibility and a possible higher mortality rate, while the B.1.351 variant has shown a reduction in the efficacy of neutralizing antibodies.
Zheng Yao Low	2022	SARS-CoV-2 Non-Structural Proteins and Their Roles in Host Immune Evasion	The article does not provide gender- specific information in relation to COVID-19.	The study outlines several mechanisms through which non-structural proteins (NSPs) like NSP1, NSP3, NSP5, and NSP6 help SARS-CoV-2 evade the immune system. These proteins interfere with immune signaling pathways by inhibiting interferon (IFN) production and altering the host's antiviral response. NSPs interact with host proteins, such as RIG-I and MAVS, to suppress innate immunity; for example, NSP1 binds to the 40S ribosomal subunit to inhibit host mRNA translation, prioritizing viral mRNA

				translation instead. Such activities also contribute to uncontrolled cytokine production, leading to severe complications in COVID-19 patients. Quantitatively, NSP5 has been shown to reduce IFN-β production by up to 10-fold, with some SARS-CoV-2 NSPs proving more effective at immune evasion than those in SARS-CoV. Additionally, compounds like montelukast and mitoxantrone have demonstrated antiviral activity against NSPs, reducing viral protein expression and viral load in laboratory tests, highlighting their therapeutic potential.
Shilei Zhang	2022	The battle between host and SARS-CoV-2: Innate immunity and viral evasion strategies	It is mentioned that mutations in the gene that encodes the TLR7 receptor, which is located on the X chromosome, have been associated with severe cases of COVID-19 in men.	The virus is recognized by pattern recognition receptors (PRRs), triggering interferon (IFN) and cytokine production. SARS-CoV-2 employs multiple strategies to evade detection, such as altering RNA to avoid host sensors and inhibiting IFN-inducing pathways. Viral proteins, like envelope (M), nucleocapsid (N), and ORF6, interfere with IFN signaling, reducing antiviral response by affecting transcription factors IRF3 and STAT1/STAT2. Also, SARS-CoV-2 infection can trigger excessive inflammation or a "cytokine storm," worsening COVID-19 severity, with ORF3a and N proteins activating the NF-kB pathway, which is linked to severe inflammation. Antiviral IFN-stimulated genes (ISGs), like CH25H, were identified as potentially inhibiting virus entry.
Gu W	2022	The molecular mechanism of SARS- CoV-2 evading host antiviral innate immunity	Comorbidities like cardiovascular disease, diabetes, and obesity, prevalent in certain groups, are linked to increased disease severity. Men generally experience more severe disease and higher mortality than women, possibly due to immune response	Monocytes, macrophages, and neutrophils in infected patients, especially in severe cases, indicating heightened inflammation. Higher ACE2 receptor expression in adults and adolescents correlates with infection susceptibility, while a decrease in CD8+ and CD4+ T cells in COVID-19 patients, particularly in severe cases, shows an impact on adaptive immunity.

			variations and comorbidity prevalence.	
Fionda C.	2022	Age-dependent NK cell dysfunctions in severe COVID-19 patients.	Adult population divided by 2 age groups (under 65, 65 and older). Older age was associated with severity and anti-viral compromised immune response.	Analyzing NK cell behavior in COVID-19 patients, significant differences were observed between adults (under 65) and the elderly (65 and older). Adults showed a substantial reduction in the total number of NK cells and specific subpopulations, such as CD56^high and CD56^low CD16^+ cells. Elderly patients had an unchanged total NK cell count but a unique subpopulation distribution, with increased CD56^low CD16^high and CD56^neg cells. Notably, IFN-g production was compromised in adults but remained robust in the elderly during early hospitalization. Hospitalization durations differed markedly, with adults averaging 15.5 days and elderly patients 33.5 days; half of the elderly required ICU treatment, and 50% died. The elderly NK cells demonstrated a more mature, activated phenotype with higher inhibitory receptor expression, indicating functional exhaustion. In contrast, adult NK cells had reduced T-BET expression, a crucial IFN-g<br>production regulator, which correlated with poorer
Sun X	2022	Immune-profiling of SARS-CoV-2 viremic patients reveals dysregulated innate immune response	Hospitalized patients with severe COVID-19 were associated with inflammation and NK cell dysfunction.	The study reveals key insights into the immune response and inflammation in COVID-19 patients. NK cell dysfunction was noted in severe cases, highlighting their critical role in immunity. Proinflammatory cytokines like IL-6 and TNF were linked to disease severity, indicating an unbalanced inflammatory response. Severe COVID-19 patients also showed reduced type I interferon production, contributing to increased viral replication. Age- related differences in NK cell function suggest age influences immune response.
Jakobs K	2022	Disease Severity in Moderate-to-Severe COVID-19 Is	Male population was more frequent in both moderate and severe clinical groups.	Patients with COVID-19 demonstrated significantly higher platelet reactivity to TRAP, ADP, and arachidonic acid

		Associated With Platelet Hyperreactivity and Innate Immune Activation		compared to those without the virus. This increased reactivity was evident in greater platelet aggregation. The SOFA score, which measures disease severity, was notably higher in COVID-19 patients, with deceased individuals showing even higher scores than survivors. Inflammatory cytokines such as IL- 6, IL-1RA, MCP-1, and CXCL10 were found at more than double the levels in COVID-19 patients compared to non-COVID-19 patients, with non- survivors exhibiting particularly high levels. In COVID-19 patients are associated with severe inflammation and worse clinical outcomes, suggesting that platelet activation plays a role in increasing morbidity and mortality. The increased formation of platelet-leukocyte aggregates indicates a significant interaction between the immune system and platelets, potentially exacerbating inflammation and clotting.
Hoste L	2022	TIM3+ TRBV11-2 T cells and IFNγ signature in patrolling monocytes and CD16+ NK cells delineate MIS-C	The pediatric population with an average of 10 years old with multisystemic inflammatory syndrome in children (MIS-C), adult population classified as moderate and severe.	Clinical Features: Patients had high fever, gastrointestinal and cardiovascular symptoms, with some neurological and respiratory signs. Distinct immune activation with patrol monocytes and high IFNγ, unlike adult COVID-19. Inflammation affected vascular and gastrointestinal tissues. Elevated proinflammatory cytokines (IL-1β, IL-6, IL-10, TNFα) and tissue damage markers. IFNγ is linked to granzyme B in NK cells. Demographics: 14 patients, average age 10, equal gender ratio. All had fever; 80% had anti-SARS- CoV-2 antibodies. Lab Markers: High ferritin, CRP, D-dimers, and neutrophils.
Hanna Kim	2022	SARS-CoV-2 peptides bind to NKG2D and increase NK cell activity	No specific population.	Two peptides (cov1 and cov2) were identified that bind to the NKG2D receptor on NK cells, suggesting a direct interaction between Peptides increase the cytotoxicity of NK cells to lung cancer cells and stimulate the production of interferon gamma (IFN-γ).

				Signaling: The activation of NK cells by peptides was mediated through the phosphorylation of Vav1, a key component in NKG2D receptor signaling.
Hammer Q	2023	NK cells in COVID-19- from disease to vaccination.	Adult hospitalized patients with clinically moderate to severe disease, and vaccinated participants.	NK cells were rapidly activated, comprising 35% of lymphocytes, although their numbers significantly decreased in severe cases. Common symptoms included fever (98%), cough (59%), and fatigue (69%). Infected cells evaded immune responses by reducing ligands for NK cell activating receptors. NK cell cytokine secretion was crucial for suppressing viral replication. mRNA vaccination increased NK cell frequency, correlating with higher antibody titers. However, in severe COVID- 19, NK cell hyperactivation could lead to inflammation and tissue damage.
Nasrollahi H	2023	Immune responses in mildly versus critically ill COVID-19 patients.	Hospitalized Adult Patients-Mild and Severe Symptoms	Interferons (IFNs), when used early in combination with other drugs, have been shown to reduce symptoms and hospital stays in mild cases, though they may also lead to adverse effects and inflammation. The immune response to SARS-CoV- 2 involves chronic T cell activation, which can result in post-COVID complications. Long-term follow-up is crucial to understand virus-host interactions and prevent severe disease progression.
